# Movement disorders in hereditary spastic paraplegias

**DOI:** 10.1055/s-0043-1777005

**Published:** 2023-11-30

**Authors:** Jose Luiz Pedroso, Thiago Cardoso Vale, Julian Letícia de Freitas, Filipe Miranda Milagres Araújo, Alex Tiburtino Meira, Pedro Braga Neto, Marcondes C. França, Vitor Tumas, Hélio A. G. Teive, Orlando G. P. Barsottini

**Affiliations:** 1 Universidade Federal de São Paulo, Departamento de Neurologia, São Paulo SP, Brazil.; 2 Universidade Federal de Juiz de Fora, Hospital Universitário, Departamento de Clínica Médica, Serviço de Neurologia, Juiz de Fora MG, Brazil.; 3 Universidade de São Paulo, Faculdade de Medicina de Ribeirão Preto, Departamento de Neurociências Comportamental, Ribeirão Preto SP, Brazil.; 4 Universidade Federal da Paraíba, Departamento de Medicina Interna, Serviço de Neurologia, João Pessoa PB, Brazil.; 5 Universidade Federal do Ceará, Departamento de Medicina Clínica, Divisão de Neurologia, Fortaleza CE, Brazil.; 6 Universidade Estadual do Ceará, Centro de Ciências da Saúde, Fortaleza CE, Brazil.; 7 Universidade Estadual de Campinas, Departamento de Neurologia, Campinas SP, Brazil.; 8 Universidade Federal do Paraná, Curitiba PR, Brazil.

**Keywords:** Spastic Paraplegia, Hereditary, Movement Disorders, Dystonia, Parkinsonian Disorders, Tremor, Paraplegia Espástica Hereditária, Transtornos dos Movimentos, Distonia, Transtornos Parkinsonianos, Tremor

## Abstract

**Background** Hereditary or familial spastic paraplegias (SPG) comprise a group of genetically and phenotypically heterogeneous diseases characterized by progressive degeneration of the corticospinal tracts. The complicated forms evolve with other various neurological signs and symptoms, including movement disorders and ataxia.

**Objective** To summarize the clinical descriptions of SPG that manifest with movement disorders or ataxias to assist the clinician in the task of diagnosing these diseases.

**Methods** We conducted a narrative review of the literature, including case reports, case series, review articles and observational studies published in English until December 2022.

**Results** Juvenile or early-onset parkinsonism with variable levodopa-responsiveness have been reported, mainly in SPG7 and SPG11. Dystonia can be observed in patients with SPG7, SPG11, SPG22, SPG26, SPG35, SPG48, SPG49, SPG58, SPG64 and SPG76. Tremor is not a frequent finding in patients with SPG, but it is described in different types of SPG, including SPG7, SPG9, SPG11, SPG15, and SPG76. Myoclonus is rarely described in SPG, affecting patients with SPG4, SPG7, SPG35, SPG48, and SPOAN (spastic paraplegia, optic atrophy, and neuropathy). SPG4, SPG6, SPG10, SPG27, SPG30 and SPG31 may rarely present with ataxia with cerebellar atrophy. And autosomal recessive SPG such as SPG7 and SPG11 can also present with ataxia.

**Conclusion** Patients with SPG may present with different forms of movement disorders such as parkinsonism, dystonia, tremor, myoclonus and ataxia. The specific movement disorder in the clinical manifestation of a patient with SPG may be a clinical clue for the diagnosis.

## Resumo

**Antecedentes** As paraplegias espásticas hereditárias ou familiares (SPG) compreendem um grupo de doenças geneticamente e fenotipicamente heterogêneas caracterizadas por degeneração progressiva dos tratos corticospinais. As formas complicadas evoluem com vários outros sinais e sintomas neurológicos, incluindo distúrbios do movimento e ataxia.

**Objetivo** Resumir as descrições clínicas de SPG que se manifestam com distúrbios do movimento ou ataxias para auxiliar o clínico na tarefa de diagnosticar essas doenças.

**Métodos** Realizamos uma revisão da literatura, incluindo relatos de casos, séries de casos, artigos de revisão e estudos observacionais publicados em inglês até dezembro de 2022.

**Resultados** O parkinsonismo juvenil ou de início precoce com resposta variável à levodopa foi relatado principalmente em SPG7 e SPG11. A distonia pode ser observada em pacientes com SPG7, SPG11, SPG22, SPG26, SPG35, SPG48, SPG49, SPG58, SPG64 e SPG76. O tremor não é um achado frequente em pacientes com SPG, mas é descrito em diferentes tipos de SPG, incluindo SPG7, SPG9, SPG11, SPG15 e SPG76. A mioclonia é raramente descrita em SPG, afetando pacientes com SPG4, SPG7, SPG35, SPG48 e SPOAN (paraplegia espástica, atrofia óptica e neuropatia). SPG4, SPG6, SPG10, SPG27, SPG30 e SPG31 podem raramente apresentar ataxia com atrofia cerebelar. E SPG autossômico recessivo, como SPG7 e SPG11, também pode apresentar ataxia.

**Conclusão** Indivíduos com SPG podem apresentar diferentes formas de distúrbios do movimento, como parkinsonismo, distonia, tremor, mioclonia e ataxia. O distúrbio específico do movimento na manifestação clínica de um paciente com SPG pode ser uma pista clínica para o diagnóstico.

## INTRODUCTION

Hereditary or familial spastic paraplegias (SPG) comprise a group of genetically and phenotypically heterogeneous diseases characterized by progressive degeneration of the corticospinal tracts. 1 They are rare diseases with an average prevalence between 1 to 5 cases per 100,000 inhabitants, affecting various ethnic groups. 1 SPG have marked genetic variability, with more than 80 genes associated with the disease, and encompassing all possible forms of genetic inheritance with autosomal dominant transmission being the most common. Symptoms usually begin insidiously at any age, more often in childhood or early adulthood, and gait disturbance is the most common initial symptom. Pure forms of SPG evolve with progressive lower-limb spasticity, urinary dysfunction, and vibratory and proprioceptive sensory changes. On the other side, the complicated forms evolve with other various neurological signs and symptoms, including movement disorders and ataxia. 1 These two may serve both as a warning for the etiologic diagnosis or as a confounder for the clinician. Apart from the family history and clinical features of progressive spastic paraparesis, the diagnosis of SPG can be supported by some magnetic resonance imaging (MRI) findings such as thinning of the spinal cord, atrophy of the corpus callosum, and white matter signal changes. However, the definitive diagnosis requires molecular genetic testing. In this instructive review, we intend to summarize the clinical descriptions of SPG that manifest with movement disorders or ataxias to assist the clinician in the task of diagnosing these diseases.

## METHODS

We conducted a narrative review of the literature in five electronic databases: PubMed, Embase, Scopus, Scielo, and Lilacs. The following search terms were used: “movement disorders” or “chorea” or “dystonia” or “myoclonus” or “tremor” or “parkinsonism” or “dyskinesia” or “tics” or “ataxia”, combined with “hereditary spastic paraplegia” or “hereditary motor sensory neuropathy”, using their respective variations according to MeSH, DECs and Emtree terms. The search limits were set to include only articles in English, published in peer-reviewed journals, during any period, limited to December 2022. We included case reports, case series, review articles, and observational studies.

## MOVEMENT DISORDERS IN SPG

Movement disorders were described in SPG patients with autosomal dominant (AD), recessive (AR), and X-linked inheritances. They can be the presenting symptom of the disease, instead of the spastic gait disturbance that is the most common initial symptom of the disease. Dystonia, tremor, parkinsonism, choreoathetosis, facial dyskinesias, myoclonus, and paroxysmal dyskinesias were all reported in association with SPG ( Table 1 ).

**Table 1:** Movement disorders associated to hereditary spastic paraplegia (SPG)

Movement disorder	HSP	Gene	Inheritance
**Ataxia**	SPG4	*SPAST*	AD
	SPG6	*NIPA1*	AD
	SPG7	*SPG7*	AD
	SPG10	*KIF5A*	AD
	SPG11	*KIAA1840*	AR
	SPG27	*10q22.1-q24.1*	AR
	SPG30	*KIF1A*	AR
	SPG31	*REEP1*	AD
**Dystonia**	SPG7	*SPG7*	AD
	SPG11	*SPG11*	AR
	SPG22	*SLC16A2*	XLR
	SPG26	*B4GALNT1*	AR
	SPG35	*FA2H*	AR
	SPG48	*AP5Z1*	AR
	SPG49	*TECPR2*	AR
	SPG58	*KIF1C*	AR
	SPG64	*ENTPD1*	AR
	SPG76	*CAPN1*	AR
**Myoclonus**	SPG4	*SPAST*	AD
	SPG7	*SPG7*	AD
	SPG35	*FA2H*	AR
	SPG48	*AP5Z1*	AR
**Parkinsonism**	SPG7	*SPG7*	AD
	SPG4	*SPAST*	AD
	SPG11	*KIAA1840*	AR
	SPG15	*ZFYVE26*	AR
	SPG48	*AP5Z1*	AR
**Tremor**	SPG7	*SPG7*	AD
	SPG9	*ALDH18A1*	AR
	SPG11	*SPG11*	AR
	SPG15	*ZFYVE26*	AR
	SPG76	*CAPN1*	AR

Abbreviations: AD, Autossomal dominant; AR, Autossomal recessive; XLR, X-linked recessive.

### Parkinsonism



**Video 1** Movement disorders in hereditary spastic paraplegia (SPG). Patients with SPG showing postural tremor (SPG11), left-sided hand tremor during walking (SPG11), upper limb dystonia (SPG46), upper-limb myoclonus (SPG78), levodopa-responsive parkinsonism (SPG7), bilateral upper-limb dysmetria (SPG7) and focal dystonia (SPG7).


Parkinsonism has been reported in SPG patients and the clinical presentation can be either tremor-dominant or rigid-akinetic, and can also be asymmetrical. Most of these patients are classified as juvenile or early-onset parkinsonism. Some have striatal dopaminergic denervation on brain SPECT and the response to levodopa treatment is variable, but some cases show good clinical response to treatment, such as in SPG11 2 3 4 and SPG7, 5 including with the development of levodopa-induced dyskinesias.

However, among the most common AD SPG, parkinsonism is not usually reported. Indeed, in the most recent genetic, structural, and clinical analysis of 157 SPG4 patients from 65 Canadian families carrying 41 different *SPAST* mutations, none had parkinsonism. 6 In a Taiwanese clinical and genetic study of 18 SPG3A patients from 11 families, none had parkinsonism. 7 In a smaller study of two unrelated adults with SPG3A using positron emission tomography, nigrostriatal dopaminergic circuitry was intact. 8 However, in an Italian cross-sectional retrospective study of SPG4 involving 723 patients from 316 families, 26.6% developed a complex phenotype, with only two patients presenting with parkinsonism. 9

The prevalence of parkinsonism in AR SPG seems to be higher in SPG7 ( Video 1 ). In a large European multicenter study involving 241 patients with SPG7, almost 7% of patients had parkinsonism within the first ten years of the disease and this prevalence reduced to only 2% within 20 years of the onset. In the same study, parkinsonism was present in different genotypes: 6% in homozygous missense mutation, 4% in heterozygous missense loss of function mutation, and 3% in homozygous loss of function mutation. 10 In a Spanish cohort of 35 patients carrying homozygous or compound heterozygous pathogenic variants in the *SPG7* gene, parkinsonism was reported in 21% of patients, with half of those responding to levodopa. 5 In Brazil, Pedroso et al. 11 have described symmetric parkinsonism with two variants of the *SPG7* gene that also responded to levodopa. The same occurred in India, 12 but unresponsive to levodopa, with improvement with pramipexole. The exact cause of parkinsonism in *SPG7* mutation is not clear, but mitochondrial dysfunction and oxidative stress seem to play a key role. The mutation of the *SPG7* gene triggers the accumulation of mitochondrial DNA (mt DNA) deletions mainly in the dopaminergic neurons of the substantia nigra and this abnormality might affect the basal ganglia. 12

SPG11, the most common AR SPG with thinning of the corpus callosum, can present with parkinsonism. Biallelic mutations in the SPG11 gene can cause juvenile-onset parkinsonism 3 13 14 15 and this finding has been confirmed in neuroimaging and neuropathological studies. One of the largest SPG11 cohorts reported on to date has described parkinsonian features in five of 30 cases, 2 60% of whom were responsive to levodopa. Other large series, however, did not report parkinsonism. 16 17 In a Brazilian cohort of 22 patients with SPG11, of whom six (27.3%) developed parkinsonism, authors showed a universal reduced dopamine transporter density. Nigral degeneration was symmetrical and correlated with disease duration and motor and cognitive handicap. 18 In another Brazilian cross-sectional case-control study 19 involving 84 patients with five different SPG subtypes analyzed with high-resolution brain T1 and diffusion tensor image, the authors showed that the SPG11 group had the most widespread pattern of brain abnormalities. Cortical thinning was identified in the basal ganglia and substantia nigra. This is similar to that reported by Faber et al., 20 explaining the dramatic phenotypic heterogeneity in the disease, including movement disorders. A Japanese neuropathological study of two patients with SPG11 showed depigmentation of the substantia nigra and their findings that motor neuron degeneration and parkinsonism in SPG11 patients is related to either TDP-43 pathology or TDP-43–negative neuronal cytoplasmic inclusions. 21

SPG11 share an overlapping phenotype with SPG15. 22 Levodopa-responsive parkinsonism has been described as the first symptom of the disease both in patients with SPG11 and with SPG15. It can also be observed during the course of the disease, usually associated with the typical signs of SPG. 13 14 23 24 Elleuch et al. 25 reported three large consanguineous Arab families with SPG15 and emphasized its clinical variability involving spastic paraplegia, saccadic pursuit and cognitive impairment, cerebellar signs, axonal peripheral neuropathy, extrapyramidal signs, and white matter abnormalities. SPG48 patients have some clinical features similar to those of SPG11 or SPG15 patients, including spastic paraplegia, retinal abnormalities, and parkinsonism. 26

### Dystonia

Dystonia can be observed in patients with SPG7, SPG11, SPG22, SPG26, SPG35, SPG48, SPG49, SPG58, SPG64 and SPG76, and in *VPS13D* mutation. SPG7, one of the most common AR SPG, can present focal or multifocal dystonia in some patients 27 28 ( Video 1 ). Dopa-responsive dystonia associated with spasticity and parkinsonism was present in a patient with SPG11, who soon developed a wearing-off phenomenon and levodopa-induced dyskinesias. The DaT-SPECT imaging indicated pre-synaptic dopamine neuronal dysfunction and the dystonia improved with a globus pallidus interna deep brain stimulation surgery. 29 Other cases of SPG11 presented with focal dystonia (toes, hands, facial, tongue, and laryngeal) and dystonia during the off period. 29 SPG22, also known as Allan-Herndon-Dudley syndrome, one of the first X-linked mental retardation syndromes reported, is a severe infantile disorder presenting with intellectual deficiency, hypotonia, progressive spasticity, associated with ataxia and/or dystonia (including paroxysmal dystonia), and with abnormalities on the thyroid hormones (increased T3, normal to mildly increased TSH, and low T4) and hypomyelination on neuroimaging. 30 SPG26 is caused by a mutation in the b-1,4-N-acetyl-galactosaminyl transferase 1 ( *B4GALNT1* ) gene, involved in the biosynthesis of glycosphingolipids, components of the synaptic plasma membrane, crucial for the central nervous system development. This disorder presents in early infancy and is characterized by slowly progressive spastic paraplegia with intellectual disability (100%), complicated by cerebellar ataxia (55%), peripheral neuropathy (60%), and facial dyskinesia and dystonia (44%). Neuroimaging usually shows cortical atrophy and white matter hyperintensities. 31

SPG35 is spastic paraplegia associated with intellectual disability, well-controlled generalized seizures, leukodystrophy, and foot dystonia beginning between ages six and 11. Dystonia could also involve the trunk, limbs, and face and leads to dysarthria and dysphagia. Homozygous mutations in the fatty acid 2-hydroxylase gene ( *FA2H* ) leads to dysmyelinogenesis. 32 SPG48 is a lysosomal storage disorder caused by biallelic mutations in the *AP5Z1* gene, which encodes the AP-5 subunit. The clinical spectrum is formed by prominent spastic paraparesis, dystonia, sensory and motor neuropathy, ataxia, myoclonus, and parkinsonism. Neuroimaging shows periventricular white matter hyperintensities (sometimes similar to the “ears of the lynx” sign), putaminal rim hyperintensity, focal atrophy of the body of the corpus callosum, and the “hummingbird sign” 33 . SPG49 is an AR SPG frequently involving the upper limbs and rarely associated with dystonic postures and cognitive alterations. The thin corpus callosum, calcification of the basal ganglia, and the ‘‘ear of the lynx’' sign can be found in neuroimaging. The disorder is caused by a mutation in the *CYP2U1* gene, which encodes an enzyme involved in fatty-acid metabolism. 34

In the SPG58, a compound mutation in the kinesin ( *KIF1C* ) gene is implicated, and the clinical picture is represented by SPG complicated by cerebellar ataxia and dystonia. Neuroimaging shows a T2-hyperintense signal in the cerebellum, pyramidal tracts, and occipital white matter with relative sparing of the optic radiations and the superior cerebellar peduncles. 35 Only a few families have been diagnosed as having SPG64, which presents as an AR early-onset SPG with cognitive impairment, dysarthria/anarthria, dystonia, and areflexia. The disease is caused by a mutation in the ectonucleoside triphosphate diphosphohydrolase 1 ( *ENTPD1* ) gene. Neuroimaging shows only mild white matter changes in some cases. 36 Garcia-Berlanga et al. 37 described a case of a patient with SPG76 with SPG complicated by oculomotor abnormalities, ataxia, bradykinesia, and cervical dystonia, in which brain MRI was unrevealing and mutation in the *CAPN1* was found. SPG78 is another complicated form of AR SPG with intellectual disability, cognitive decline, psychosis, upward ophthalmoplegia, neuropathy, dystonia, and thin corpus callosum. 38

### Tremor

Tremor is not a frequent finding in patients with SPG, but it is described in different types of SPG, including SPG7, SPG9, SPG11, SPG15 and SPG76 ( Video 1 ). Recently, Kalmar et al. 39 described a patient with *ALDH18A1* gene mutation (SPG9) with early-onset tremor, preceding lower limb spasticity. SPG11 patients may have a complex phenotype including dopa-responsive dystonia and tremor. Innes et al. 40 described a case of SPG11 presenting as childhood-onset dystonic tremor without weakness or spastic paraplegia. Patients with SPG7 may have complex or mixed phenotype including the presence of parkinsonism, dystonia, and tremor. Atypical presentation of SPG7, such as the presence of palatal myoclonus, described by Primiano et al. 41 are increasingly common in the literature. Alecu et al. 42 described an 18-year-old patient with *CAPN1* missense mutation (SPG76) who presented with psychiatric symptoms followed by spastic gait, intention tremor, and neurogenic bladder dysfunction. Ersen et al. 43 reported a case of a SPG15 patient with topiramate-responsive tremor. There are rarely described cases of orthostatic tremor associated with SPG, one of them with improvement after the use of levodopa. 44 45 In summary, there are reports that patients with SPG may present with tremors, including postural, task-specific resting, or kinetic tremors. 43 46 47 48 Descriptions regarding the treatment of these patients with SPG and tremors are scarce.

### Myoclonus

Myoclonus is rarely described in SPG. Mutations in *AP5Z1* , a gene playing a role in intracellular membrane trafficking, have been reported to be associated with SPG48 with a diverse spectrum of movement disorders, including ataxia, myoclonus, spasticity, dystonia, and parkinsonism. 33 Although not an SPG, the adult form of Alexander's disease should be included in the differential diagnosis. Patients usually present palatal myoclonus with pyramidal tract signs like spasticity. The diagnosis of Alexander's disease is established with a heterozygous pathogenic variant in *GFAP* identified by molecular genetic testing. 49 There are rare cases described of non-epileptic myoclonus, affecting patients with SPG4, SPG7, SPG35, SPG48, and SPOAN (spastic paraplegia, optic atrophy, and neuropathy). 5 33 50 51 52

### Other movement disorders in SPG

In some rare cases of SPG, orofaciobucolingual dyskinesias have been reported 31 53 54 as well as some cases of generalized chorea. 51 There are reports of paroxysmal non-kinesiogenic dyskinesias in a patient with mutations in *SLC16A2* gene and exercise-induced dyskinesias in a patient with SPG8. 55 56

Mutation in the *VPS13D* causes an early-onset SPG complicated by cerebellar ataxia, cervical dystonia, and some patients with chorea and tremors. 57 Oromandibular akinetic mutism, dyskinesia, and athetoid movements of the extremities were described in patients with SPG21 (also known as Mast syndrome) in advanced stages, when patients are typically demented. Brain MRI shows a thin corpus callosum and white-matter abnormalities. 53 A dominantly inherited SPG is caused by heterozygous mutation in *ATAD3A* , which can mimic dyskinetic cerebral palsy with developmental delay, hypotonia, spasticity, optic atrophy, axonal neuropathy, and cardiac abnormalities. 58

Oculomotor changes, such as ptosis, saccadic pursuit impairment, vertical gaze limitation, and nystagmus can occur in some of the complex SPG, though none of these changes are highly suggestive of a specific subtype. In an SPG7 cohort, oculomotor examination showed asymmetric ptosis, saccadic pursuit, and a limitation of vertical gaze. 10 In another SPG7 cohort of 35 individuals, progressive external ophthalmoplegia, nystagmus, ptosis, and saccadic intrusions were reported, respectively, in nine, seven, five, and four patients. 5

In a Canadian cohort of 157 SPG4 patients from 65 families with different mutations, only one patient had oculomotor abnormalities, involving hypometric saccades, pursuit smooth impairment, and mild horizontal gaze-evoked nystagmus. 6 In two SPG5A patients out of 105 Italian probands with pure or complex SPG, Arnoldi et al. 59 identified a complicated form with nystagmus, dysarthria, and sensorineural hearing loss in one and cataract and mild cognitive impairment in the other. Mukai et al. 60 have reported on two sisters with SPG11 mutation with a clinical picture resembling multiple sclerosis. One of the sisters had bilateral oculomotor disturbance twice and the examination showed saccadic eye movements. The same occurred with a Japanese man with SPG11 mutation. 21 As mentioned previously, SPG11 and SPG15 mutations share pathophysiological mechanisms and usually lead to a complex phenotype including cerebellar findings, such as ataxia and impaired extraocular muscle movements. Not surprisingly, ataxia with oculomotor apraxia type 2 is one of the differential diagnoses of SPG with complex phenotype involving the oculomotor system.

### Ataxia

The group of diseases which share spastic paraplegia and ataxia as clinical manifestations are usually called spastic ataxia. Hereditary spastic ataxias comprise a very large differential diagnosis which includes AD ataxias, AR ataxias, and SPG with the combined phenotype of ataxia and spastic paraplegia. 16 61 The variability of phenotypes observed with the traditionally called hereditary ataxias and SPG group of diseases warrants a rethinking of the traditional classification system. 62 We will discuss the main causes of SPG that have also ataxia in its clinical spectrum.

SPG4, related to *SPAST* gene mutation, is the most common SPG and may rarely present with cerebellar atrophy. 63 Other AD SPG may also present with ataxia, including SPG6, SPG10, SPG27, SPG30 and SPG31. 61 Moreover, another AD neurologic disorder that manifests either as isolated spastic paraplegia or the combination of ataxia, spastic paraplegia, and mental retardation, was described in 2002 and named as spastic paraplegia, ataxia and mental retardation (SPAR). 64

AR subtypes of SPG may also present ataxias as a common feature. Of note, the SPG7 phenotype may vary between pure spastic paraplegia and a predominantly spastic-ataxic phenotype with cerebellar atrophy on brain MRI. 65 Less frequently, SPG11 can present with ataxia, but usually, patients also have mild intellectual disability or progressive cognitive decline and peripheral neuropathy, with brain MRI typically disclosing tinning of the corpus callosum. 66

In conclusion, patients with SPG may present with different forms of movement disorders such as parkinsonism, dystonia, tremor, myoclonus, and ataxia. The specific movement disorder in the clinical manifestation of a patient with SPG may be a clinical clue for the diagnosis. Also, the definition of the phenomenology of the movement disorder may guide specific treatments, such as levodopa use, botulinum toxin, and others.
